# Supplementary material for: Chromothripsis during telomere crisis is independent of NHEJ, and consistent with a replicative origin
Source: Genome Res. 2019 May;29(5):737–49. doi: 10.1101/gr.240705.118 (PMC6499312; doi:10.1101/gr.240705.118)
Supplement: Supplemental Material [file supp_gr.240705.118_Supplemental_file_1.zip › contigs/annotated_contigs/DB50/contig.2.DB50_length_637_mean_cov_9.52904238619.docx]

**DB50_length_637_mean_cov_9.52904238619**

```
CACAATCACTCCAGAAATGAGAAATCAGGAGAGACGTCCTGGAGACAGGGAACTTCAGGTGGTCTTTGAAATCTGTTCAGTGTTGAAGC
 >chr10:1354457-1354735 - E=1e-150
CAAAATGTGATGAAGTGAAGAGAAACTCACATTCAGGCCACAGCAGAATAACTGAACCTGCTGCATCAGCCTCAGAGCCAGCCTGGGGC

CTGCCGGGGGCTCACTCTGACCTGGAGGTGGCCACAGGAGCTGCTGGAAGAAGGTATGGCTGTTGCCCTTTCTTCCAGAAAGTGCATCA

CCCCCACACCC|TG|ATTTCACCAAAGTCTCTATGATTTCCTGCTGCTTTGTTTATTAAGTCACTCATCAGATGTTGAATGGGGCCTAT
            >chr10:1352273-1352632 + E=3e-201
TTTGGACCGAGCACTGTGCCAGGTGCTGCTGTCACACTGAGTGAGCAGTCTGAAGGTGTTTGCATTTATGCAGAGGTACAACTTCTGAC

TTCCACCATGCCTGGCACTGAGAGACACTCCCAGATGTTAAAATATTCACCAGTCCTGGAGAAGCAGCTGGCACTGAGGCTCAGCACTC

CTGCTAACCTACGAAGATGTTGGAATCAATGAGAAAAACTTCATTGACTCTGAATAACATTCCTTCATTGACTCTGAATAACATTCCTT

CATTGACTCTGAATAA
```
